# AMPK/SIRT1 Pathway is Involved in Arctigenin-Mediated Protective Effects Against Myocardial Ischemia-Reperfusion Injury

**DOI:** 10.3389/fphar.2020.616813

**Published:** 2021-01-26

**Authors:** Cheng-Yin Liu, Yi Zhou, Tao Chen, Jing-Chao Lei, Xue-Jun Jiang

**Affiliations:** ^1^ Department of Cardiology, Renmin Hospital of Wuhan University, Wuhan, China; ^2^ Cardiovascular Research Institute, Wuhan University, Wuhan, China; ^3^ Hubei Key Laboratory of Cardiology, Wuhan, China

**Keywords:** myocardial infarction, arctigenin, AMPK/SIRT1 pathway, inflammation, oxidative stress

## Abstract

Arctigenin, one of the active ingredients extracted from Great Burdock (Arctium lappa) Achene, has been found to relieve myocardial infarction injury. However, the specific mechanism of Arctigenin against myocardial infarction remains largely unknown. Here, both acute myocardial ischemia-reperfusion injury (AMI/R) rat model and oxygen glucose deprivation (OGD)-induced myocardial cell injury model were constructed to explore the underlying role of AMPK/SIRT1 pathway in Arctigenin-mediated effects. The experimental data in our study demonstrated that Arctigenin ameliorated OGD-mediated cardiomyocytes apoptosis, inflammation and oxidative stress in a dose-dependent manner. Besides, Arctigenin activated AMPK/SIRT1 pathway and downregulated NF-κB phosphorylation in OGD-treated cardiomyocytes, while inhibiting AMPK or SIRT1 by the Compound C (an AMPK inhibitor) or SIRT1-IN-1 (a SIRT1 inhibitor) significantly attenuated Arctigenin-exerted protective effects on cardiomyocytes. In the animal experiments, Arctigenin improved the heart functions and decreased infarct size of the AMI/R-rats, accompanied with downregulated oxidative stress, inflammation and apoptotic levels in the heart tissues. What’s more, Arctigenin enhanced the AMPK/SIRT1 pathway and repressed NF-κB pathway activation. Taken together, our data indicated that Arctigenin reduced cardiomyocytes apoptosis against AMI/R-induced oxidative stress and inflammation at least via AMPK/SIRT1 pathway.

## Introduction

Myocardial infarction (MI) is the myocardial necrosis resulting from persistent coronary ischemia or hypoxia (Lu et al., 2015). Clinically, severe and persistent retrosternal pain, which is accompanied by increased serum myocardial enzyme activity and progressive electrocardiogram changes, maybe complicated with arrhythmia, heart rupture, shock or heart failure severe cases, cannot be entirely alleviated by rest and nitrates (Okano and Shiba, 2019). With the advancing of reperfusion therapies, ischemia-induced myocardial damage in acute myocardial infarction patients has been markedly reduced. However, reperfusion-induced cardiac injury has become increasingly evident (Wang et al., 2020). It is reported that the mechanism of myocardial injury induced MI is complicated, mainly related to apoptosis and necrosis of myocardial cells, inflammation and oxidative stress (Zhou et al., 2020). Among them, oxidative stress is the result of the imbalance between reactive oxygen species (ROS) production and antioxidant defense system. Studies have shown that oxidative stress makes a significant contribution to myocardial ischemic injury, and its further development can lead to myocardial remodeling (Sinha and Dabla, 2015). On the other hand, TNF-α and IL-1β exert toxic effects on the myocardium and lead to direct myocardial damage by promoting the overactivation and differentiation of macrophages and stimulating macrophages to secrete IL-6, IL-8 and other inflammatory factors (Lewis et al., 2012). Hence, it is of considerable significance to reduce oxidative stress and inflammation to treat myocardial cell injury, apoptosis and MI.

Various natural products have been identified as relevant sources for the invention of drugs against heart damage. Arctigenin (ATG, formula: C₂₁H₂₄O₆) is a natural lignan compound extracted from *Great Burdock (Arctium lappa) Achene*. Studies have revealed that ATG has anti-tumor, anti-microbial, anti-oxidation, anti-apoptosis, anti-inflammation and other functions (Lewis et al., 2012; Gao et al., 2018; Sun et al., 2018). The experimental study of Ni SH et al. found that Arctigenin improved AMI by inhibiting the inflammatory phenotype of macrophages/monocytes through targeting of NFAT5 (Ni et al., 2020). This phenomenon demonstrates that Arctigenin also has significant value in heart protection in animals. However, the underlying mechanisms of Arctigenin on OGD-induced MI have not been reported in detail.

Adenosine 5′-monophosphate (AMP)-activated protein kinase (AMPK) is an AMP-dependent protein kinase. An important kinase that regulates energy homeostasis is mainly responsible for monitoring cell input and output and maintaining the smooth operation of cell physiological activities (Hardie et al., 2016; Kim et al., 2016). Sirtuin-1 (SIRT1), a nicotinamide adenine dinucleotide-dependent deacetylase, can deacetylate histones and non-histones and other transcription factors, it participates in various physiological functions such as cell aging, gene transcription, energy balance and regulation of oxidative stress (Meng et al., 2017; Alves-Fernandes and Jasiulionis, 2019). In recent decades, mounting studies have shown that Arctigenin activates AMPK and SIRT1, thus involving in various pathophysiological processes. For example, Arctigenin reduces adipogenesis in high-fat diet-induced obese mice and then decreases weight gain of the obese mice through upregulating AMPK (Han et al., 2016). In middle cerebral artery occlusion (MCAO) rat model, Arctigenin showed neuroprotective effects against cerebral ischemia via SIRT1-dependent inhibition of NLRP3 inflammasome (Zhang et al., 2017). Besides, studies have also shown that Arctigenin can ameliorate lipopolysaccharide-induced acute lung injury in rats through modulating NF-κB p65, IκBα, and AMPKα (Shi et al., 2015). Similarly, in the rat myocardial infarction, Danqi tablets (DQT) upregulates the production of ATP in the myocardial cells of rats with ischemic heart disease through the AMPK/SIRT1-PGC-1α pathway, and inhibit the infiltration of inflammatory cells in the marginal area of MI to protect myocardium (Meng et al., 2019). However, whether Arctigenin has a significant value in MI through the AMPK/SIRT1 signaling pathway awaits further investigation.

The present study aims at the mechanism of natural drug Arctigenin in AMI/R rats. Our study results manifested that Arctigenin considerably attenuated OGD-mediated myocardial cell injury *in vitro* and promoted the activation of the AMPK/SIRT1 pathway. Therefore, we speculated that Arctigenin could play a myocardial protective role by activating the AMPK/SIRT1 signaling pathway to reduce oxidative stress and inflammation. We further explored the myocardial protective effect of Arctigenin in AMI/R rat model, hoping to provide a new theoretical reference for the treatment and prognosis improvement of MI.

## Materials and Methods

### Establishment of MI Model

Eighty adult Sprague-Dawley (SD) rats (male, 225 ± 5 g in weight) were purchased from the Animal Experiment Center of Wuhan University. Before the experiment, the animals adapted to the environment following circadian rhythms for 1 week. They were raised in Specific pathogen Free (SPF) condition: 12/12 h light/dark circle; 25 ± 2 °C temperature and 50 ± 15% humidity. These animals were free to eat and drink. Rats were randomly divided into control group (Sham group) and the I/R group. The Ethics Committee of Wuhan University People’s Hospital approved all animal experiments.

Acute myocardial ischemia-reperfusion injury (AMI/R) rat model was used for *in vivo* experiment. Before surgery, the rats were fasted for 12 h and then were anesthetized with pentobarbital sodium (50 mg/kg) by intraperitoneal injection. After anesthesia, the skin was removed from the left chest, and the rat was secured to a thermostatic controller. Then, we intubated the cannula into the trachea, connected the small animal ventilator to assist in breathing, disinfected and cut the skin between the third and fourth ribs of the left chest, separated the muscle layer, and exposed the left ventricle and left atrial appendage. Next, a small curved needle with a 6-0 silk suture was passed through the myocardium beneath LAD, and a ligation was placed to block the blood flow. The AMI/R model was successfully established when the ST segment in lead II was elevated and the regional myocardial surface that became pale was observed. Thirty minutes later, knot was untied to perfuse myocardium. At 4 h post-reperfusion, mechanical ventilation was stopped after the rats’ heart rate and respiration were stable, and the cannula was kept for further observation. When the rats were treated with thermal insulation 24 h after operation. Threading was performed on rats in the sham group without ligation; other operations were consistent with the AMI/R group.

### Myocardial Cell Culture and Drug Treatment

The hearts obtained from ten new-born (1–2 days) adult Sprague-Dawley (SD) rats were removed rapidly into cold Hanks’ balanced salt solution (HBSS). After washing and mincing, tissues were digested in 0.05% trypsin (GIBCO, United States) for 30 min at 4 °C with rotation. The tissues were transferred into DMEM (GIBCO, United States) containing 20% FBS (Fetal bovine serum, Gibco) to terminate the digestion. The supernatant containing released cells was removed every 10 min into an equal volume of cold DMEM complete medium (abandon the first two times) until most of the myocardium was disrupted. Cells were collected and enriched by two sequential pre-plating steps on 90-mm dishes which removed non-cardiomyocytes. The isolated cells were incubated with 5% CO_2_ at 37 °C for 1 h. The unattached cardiomyocytes were seeded into fibronectin-coated 12-well tissue culture plates (Costar, United States) and subsequent experiments were performed when the cardiomyocytes formed a confluent monolayer and beat in synchrony at 72 h.

The Cell Center of the Chinese Academy of Sciences (Shanghai, China) was the supplier of H9C2 cardiomyocytes. Cells were cultured with RPMI1640 (Thermo Fisher Scientific, MA, United States) containing 10% fetal bovine serum (FBS, Thermo Fisher Scientific) and 1% penicillin/streptomycin (Invitrogen, CA, United States) in an incubator (5% CO_2_, 37 °C). Cells during the logarithmic growth phase were taken and underwent 0.25% trypsinization (Thermo Fisher HyClone, Utah, United States) for generation.

Arctigenin (article number: HY-N0035), AMPK inhibitor Compound C (article number: HY-13418A) and SIRT1 inhibitor SIRT1-IN-1 (article number: HY-136199) were purchased from MedChemExpress (SH, United States). Arctigenin and Compound C were first dissolved in 10% DMSO and then diluted in 0.9% normal saline. In experiments *in vitro*, H9C2 cardiomyocytes were treated with Arctigenin (0, 25, 50, 100, 200, 400 µM) or Compound C (1 nM) or SIRT1-IN-1 (0.2 µM) in the medium for 24 h. *In vivo*, rats were treated with Arctigenin at (100 µmol/kg) through intraperitoneal injection.

### Establishment of an OGD Model

H9C2 cells and primary cardiomyocytes were exposed to hypoxia (partial oxygen pressure was maintained below 2 mmHg). Sugar-free balanced salt solution (116 mM NaCl, 5.4 mM KCl, 0.8 mM MgSO_4_, 1.0 mM NaH_2_PO_4_, 1.8 mM CaCl_2_, 26.2 mM NaHCO_3_, 0.025 mM phenol Red, and 20 mM sucrose) was substituted for H9C2 cell culture solution and placed at 37 °C. The cells were foamed with an anaerobic gas mixture (95% N_2_, 5% CO_2_) for 30 min and cultured in a solution at 37 °C for another 4 h before being reoxygenated (returning to a normal erobic environment). 12 hours after reoxygenation, the experimental parameters were measured.

### MTT Assay

After trypsinization, H9C2 cardiomyocytes and primary cardiomyocytes at the logarithmic growth stage were inoculated into 96-well plates for 24 h culture at a density of 5 × 10^3^/ml. The old culture medium was abandoned, and Arctigenin was added at different concentrations (0, 25, 50, 100, 200, 400 µM) for 24-h further culture, with 5 replicates for each experimental group. The medium containing the drug was abandoned, and 50 μL MTT solution Sigma-Aldrich (St. Louis, MO, 5 mg/ml) was added in each well for another 4 h. Then, the supernatant was discarded, and the cells were shaken for 1.5 h with the addition of 200 μL DMSO. When the cells were completely lyzed, the absorbance value was evaluated with a microplate reader at a wavelength of 450 nm.

### Flow Cytometry

H9C2 cells and primary cardiomyocytes treated with different factors underwent trypsinization and centrifugation (1,500 r/min, 3 min) for collection. The obtained cells were operated according to the instructions of the apoptosis detection kit (Shanghai Zeye Biological Reagent Company, China, Article No.: ZY140626): After washing the cells twice with PBS, we added 400 μL pre-cooled PBS. Then, 10 μL AnnexinV-FITC and 5 μL PI were added, respectively. After the 30-min incubation (4 °C, in the dark), the flow cytometry was immediately performed to examine cell apoptotic. The percentage of apoptotic cells was calculated after the computer software process. Apoptosis rate = number of apoptotic cells/(number of apoptotic cells + number of normal cells) × 100%. All the steps were in accordance with the kit instructions.

### Real-Time Polymerase Chain Reaction

Using TRIzol reagent (Invitrogen, Waltham, MA, United States), total RNA was extracted from tissues or cells. RNA concentration and purity were assessed with the Nanodrop-spectrophotometer. According to the manufacturer’s scenario, 1 µg of total RNA was reverse-transcribed using the PrimeScript-RT Kit (Madison, WI, United States) to synthesize its complementary DNA (cDNA). Then, SYBR® Premix-Ex-Taq™ (Takara, TX, United States) and the ABI7300 system were applied to perform the RT-PCR on cDNA. The PCR system was 30 µL in total volume and contained 300 ng cDNA in each sample. The amplification process was initial denaturation (10 min, 95 °C), followed by 45 cycles (95 °C, 10 s; 60 °C, 30 s; 85 °C, 20 s). We converted all fluorescence data into relative quantification and took GADPH as the endogenous control for IL-6, IL-1β and TNF-α (Table 1). All RT-PCR reactions were repeated three times. Guangzhou Ruibo company designed and synthesized the primers.

**TABLE 1:** Primer sequences of each gene.

Gene	Primer sequence (5′→3′)
TNF-α	forward: CAG​GGG​CCA​CCA​CGC​TCT​TC
	reverse: CTT​GGG​GCA​GGG​GCT​CTT​GA
IL-6	forward: ATG​AAC​TCC​TTC​TCC​ACA​AGC​GC
	reverse: GAA​GAG​CCC​TCA​GGC​TGG​ACT​G
IL-1β	forward: TCC​CTT​CAT​CTT​TGA​AGA​AGA
	reverse: GAG​GCC​CCA​AGG​CCA​CAG​G
	reverse: GGC​AAG​CCC​GTT​AAA​CTC​AA
GADPH	forward: AGA​AGG​CTG​GGG​CTC​ATT​TG
	reverse: nkAGGGGCCATCCACAGTCTTC

### Western Blot

H9C2 cells and primary cardiomyocytes were collected and washed three times with cold PBS, 100∼200 μL RIPA lysates were added (Beyotime Biotcchnology, Shanghai, China), cells were lyzed in ice water by ultrasound. Bradford method was taken to measure protein concentration. Equal amounts of protein from each group were subjected to 10% SDS-PAGE electrophoresis, and the protein on the gel was transferred to PVDF membranes (Millipore, Bedford, MA, United States). Next, these membranes were blocked for 1 h and added with the primary antibody (concentration 1:1,000), Anti-p-AMPKα antibody (ab23875), Anti-AMPKα antibody (ab32047), Anti-SIRT1 antibody (ab110304), Anti-p-NF-κB antibody (ab28849), Anti-NF-κB antibody (ab7549), Anti-pI-κB antibody (ab178846), Anti-I-κB antibody (ab230341), Anti-Caspase3 antibody (ab13847), Anti-Bcl2 antibody (ab182858) for overnight incubation (4 °C). Then, TBST was used to wash the membranes twice. They were incubated with fluorescein-labeled anti-sheep anti-rabbit (AB205718, 1:2,500) (1 h, room temperature). The above antibodies were all from Abcam, Cambridge, United Kingdom. After being washed three times, they were exposed to ECL developer (Millipore, Bedford, MA, United States) and imaged with a membrane scanner.

### Echocardiography

M-type echocardiography was conducted 1 day before the end of the experiment. The rats were anesthetized with pentobarbital sodium (50 mg/kg, i.p.) in the supine position and secured on a heating plate at 37 °C. The limb was connected to the electrocardiogram electrode to monitor the heart rate and record the electrocardiogram. Chemical depilation cream was used for depilation to reduce ultrasonic interference. The chest surface was coated with an ultrasonic guide agent to optimize the cardiac cavity environment for ultrasonic detection. Hemodynamic parameter left ventricular ejection fraction (LVEF) was recorded.

### TTC Staining

The rat was anesthetized with pentobarbital sodium (50 mg/kg, i.p.), cut off at the inferior vena cava, and the heart was removed by flushing it from the aortic arch. The residual blood was rinsed with cold PBS, and the left ventricle weight was recorded. The left ventricle was cut into slices with a thickness of about 1–2 mm, and the weight of each piece was recorded. The slices were put into the freshly prepared 2% TTC phosphate buffer and dyed, avoiding light at 37 °C. After 30 min, the slices were secured in 4% paraformaldehyde solution for preservation. The infarcted area was white, and the non-infarcted area was dark red. Image-Proplus 5.1 software (Media Cybemetics, United States) was employed for data analysis of the infarct area. Calculation of MI area: the area of the infarcted part/total area of the slice × 100%.

### ROS Detection

The levels of ROS in the cells or tissues were measured using a DCFDA Cellular ROS Detection Assay kit (cat. no. ab113851; Abcam, Cambridge, United Kingdom) according to the manufacturer’s protocols; the fold change relative to the controls was compared.

### Assessment of Oxidative Stress (OS) Level

Partial heart tissues of different groups (*n* = 5) were dissolved and homogenized with a microcentrifuge tube homogenizer, then centrifuged (5000 RPM, 15 min) to obtain the respective supernatant. As per the manufacturer’s agreement, malondialdehyde (MDA) content and superoxide dismutase (SOD) activity were evaluated in the heart homogenate supernatant using specific detection kits. Nanjing JianCheng Bioengineering Institute (China) was the supplier of the oxidative stress detection kits.

### HE Dyeing

First, the chambers were fixed in 4% formaldehyde (overnight, 4 °C). Secondly, the sample tissues went through dehydration, vitrification and paraffin-embedding. Eventually, they were made into slices 6 μm in thick. Then, we removed the paraffin using xylene, blocked off moisture by high concentration to a low concentration of ethanol, put the slices into the hematoxylin aqueous solution to dye for a few minutes, separated the color in the acid and ammonia water every few seconds, rinsed them 1 h with flowing water, and placed them in distilled water for a while. After 2∼3 min dyeing with eosin staining fluid, dehydration was performed with anhydrous ethanol. Finally, the slices were made transparent using xylene and mounted. The staining effect was observed under an inverted microscope, and the pathological changes of the tissues were observed under 100-fold and 200-fold field of vision.

### Immunohistochemistry

Conventional paraffin embedding and continuous coronal sections were carried out. Immunohistochemical staining was performed on each section with a thickness of about 5 μm. After conventional xylene dewaxing and gradient alcohol hydration, the endogenous peroxidase was inactivated by being blocked with 3% H_2_O_2_ for 10 min. Then, 0.01 mmol/L sodium citrate buffer was used for microwave repair (pH = 6.0, 15 min). The slices were blocked with 5% bovine serum albumin (BSA) for 20 min, then added with primary antibody Caspase3 (ab13847, MA, United States), AMPK (ab32047), MA, United States) (4 °C, overnight). The next day, goat antibody and rabbit secondary antibody were added. After a 20 min incubation at room temperature, the section was PBS washed and color visualized by DAB. Finally, after hematoxylin counterstaining, it was dehydrated till transparent and mount examined. Image-Pro Plus image analysis software ((Media Cybemetics, United States) was taken to analyze the number of Caspase3 positive cells and the expression of AMPK.

### Data Analysis

Data analysis was carried out via SPSS20.0 statistical software (SPSS Inc., Chicago, IL, United States). The data were presented as mean ± standard deviation (m ± SD). Univariate ANOVA was used for the multi-factor comparison, and an independent sample *t*-test was used for the comparison between two groups. *p* < 0.05 was taken as statistically meaningful.

## Results

### Arctigenin Inhibited OGD Mediated Apoptosis of Cardiomyocytes

**FIGURE 1:**
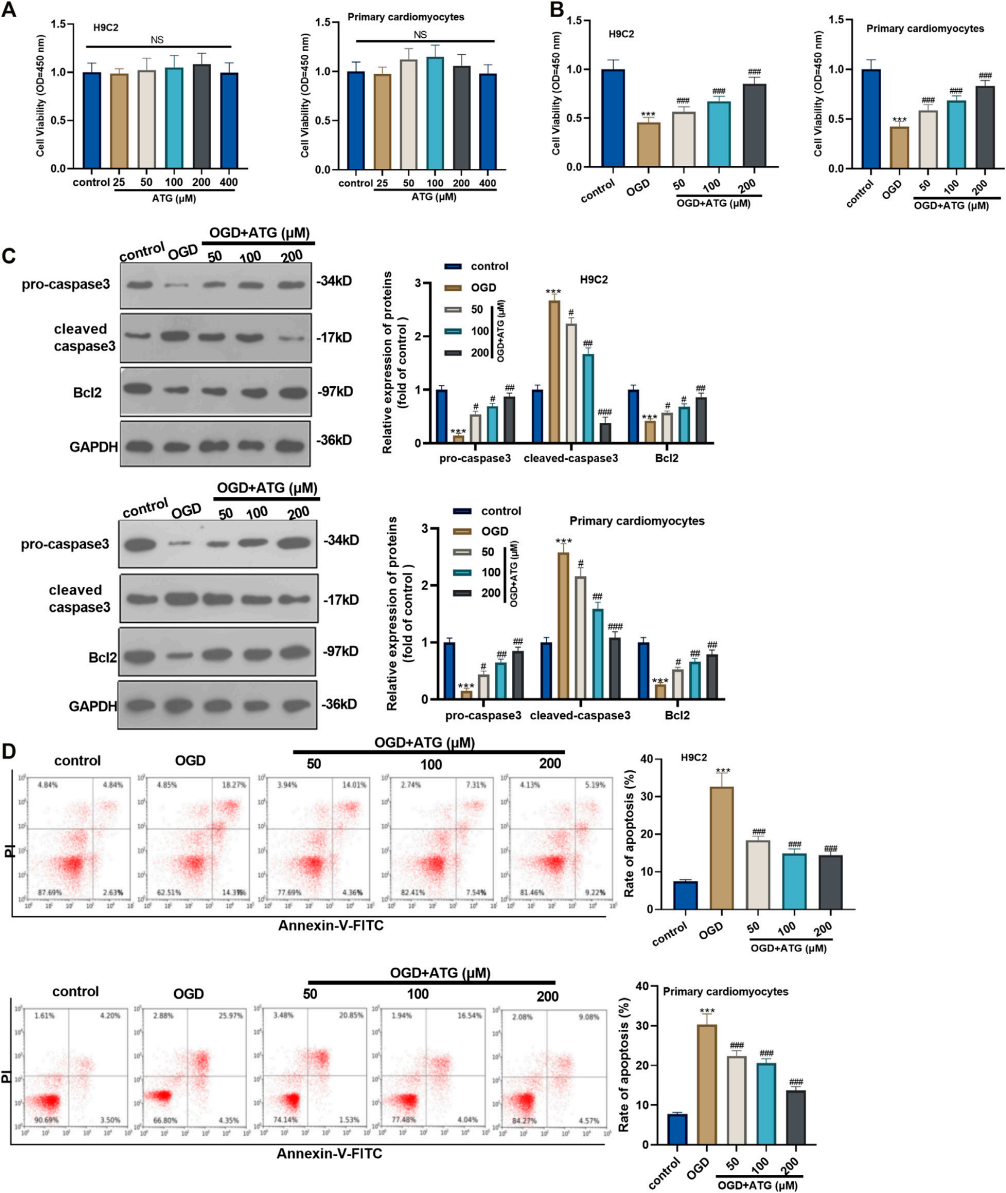
Arctigenin inhibited OGD-mediated cardiomyocyte apoptosis. An *in vitro* OGD model was constructed in H9C2 cardiomyocytes and Primary cardiomyocytes and given different concentrations (25, 50, 100, 200, 400 µM) of Arctigenin for intervention. **(A, B)** MTT method was conducted to evaluate the effect of Arctigenin on cell viability at different concentrations. **(C)** Western blot experiment was carried out. With the treatment of gracillin, the protein levels of Caspase-3 and Bcl2 were detected. **(D)** Flow cytometry was performed to measure the OGD-mediated H9C2 cell and Primary cardiomyocytes apoptosis. Ns *p* > 0.05, ***p < 0.001 (vs.control group), #*p <* 0.05, ##*p <* 0.01, ###*p* < 0.001 (vs.OGD group), *N* = 5.

First of all, H9C2 cells and primary cardiomyocytes were interfered with Arctigenin at different concentrations (25, 50, 100, 200, 400 µM) to detect its cytotoxicity. H9C2 cell and primary cardiomyocytes viability showed no significant change with Arctigenin’s interference, indicating that Arctigenin had no cytotoxicity in cardiomyocytes (*p* > 0.05, Figure 1A). Then, OGD was performed to construct a cardiomyocyte injury model *in vitro*. On this basis, H9C2 cells and primary cardiomyocytes were treated with Arctigenin at different concentrations (0, 50, 100, 200 µM). It was found that the cell viability was notably decreased after OGD treatment, while the viability was increased with the treatment of Arctigenin in a dose-dependent manner (compared with the OGD group) (*p* < 0.05, Figure 1B). Besides, Western blot experimental statistics illustrated that with the increase of Arctigenin’s concentration, Caspase 3 (apoptosis promoting protein) activity decreased, while the expression of Bcl2 (apoptosis suppressing protein) increased (*p* < 0.05, Figure 1C). Further, flow cytometry was carried out to measure apoptosis of H9C2 cells and primary cardiomyocytes, and the results revealed that compared with OGD group, Arctigenin inhibited cell apoptosis in a dose-dependent way (*p* < 0.05, Figure 1D). The above results demonstrated that Arctigenin considerably attenuated OGD-mediated myocardial cell injury.

### Arctigenin Inhibited OGD-Mediated Oxidative Stress and Inflammatory Response in Cardiomyocytes

**FIGURE 2:**
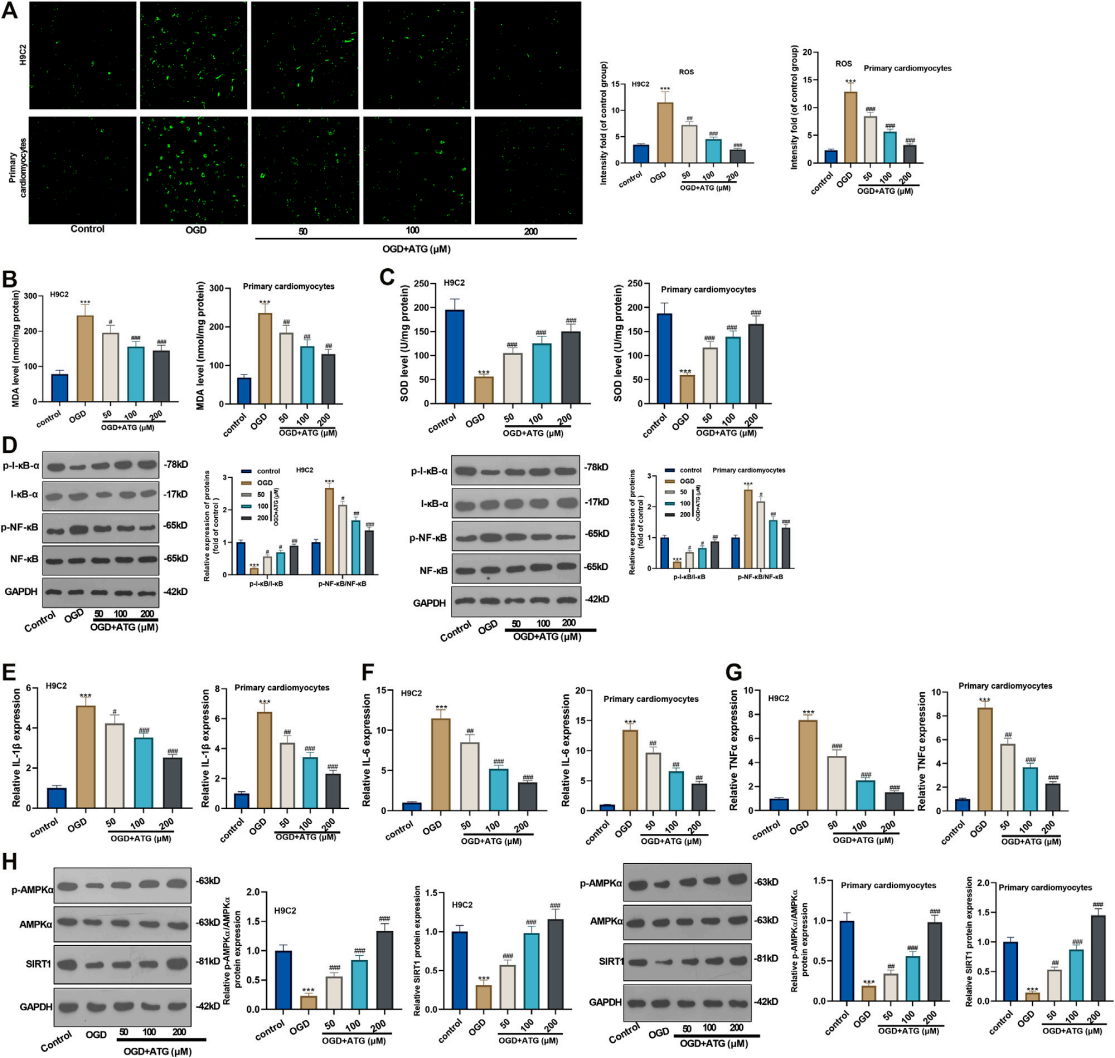
Arctigenin inhibited OGD-mediated oxidative stress and inflammation *in vitro*. On the basis of OGD treatment on H9C2 cardiomyocytes and Primary cardiomyocytes, different concentrations (50, 100, 200 µM) of Arctigenin were given for intervention. **(A)** Using DCFH-DA fluorescent dye to study Arctigenin’s effect on OGD-induced ROS levels in H9C2 cells and Primary cardiomyocytes. **(B, C)** RT-PCR experiment was taken to examine the content of MDA and SOD activity in OGD treated-cardiomyocytes. **(D–G)** Western blot and RT-PCR experiments were utilized to assess the protein expression of NF-κB/I-κB and the levels of inflammatory factors IL-6, IL-1β and TNF-α. **(H)** Western blot assay to detect the protein expression of the AMPK/SIRT1 pathway. ***p < 0.001 (vs.control group), #*p* < 0.05, ##*p* < 0.01, ###*p* < 0.001 (vs.OGD group), *N* = 5.

Excessive reactive oxygen species can lead to imbalanced redox, thus inducing oxidative stress. Hence, we initially used DCFH-DA fluorescence to study Arctigenin’s effect on the OGD-induced ROS level in H9C2 cells and primary cardiomyocytes. It turned out that OGD treatment notably increased the ROS level in H9C2 cells and primary cardiomyocytes compared with the control group. However, the ROS level decreased gradually with Arctigenin’s treatment at different concentrations, and the ROS reduction was in accordance with Arctigenin in a dose-dependent way (compared with the OGD group) (*p* < 0.05, Figure 2A). Further, we measured the oxidative stress level in the OGD damaged rat heart *in vitro*. It was found that compared with the control group, the MDA content in OGD myocardial cells was higher, and the SOD activity was lower. Interestingly, treatment with Arctigenin attenuated the MDA level while increased SOD level in H9C2 cells and primary cardiomyocytes (*p* < 0.05, Figures 2B,C). Those results suggested that Arctigenin may play a cardiac protective role by regulating oxidative stress. Next, Western blot and RT-PCR were utilized to examine the protein expression of NF-κB/I-κB and the levels of inflammatory factors (IL-6, IL-1β, and TNF-α). It turned out that compared with the control group, OGD upregulated the expression of p-NF-κB, but down-regulated the expression of p-I-κB, and the expressions of inflammatory factors were also remarkably increased (Figures 2D–G). On this basis, different concentrations of Arctigenin was added, we discovered that Arctigenin suppressed the phosphorylation level of NF-κB in a dose-dependent way, but upregulated the phosphorylation expression of I-κB, and the expression of inflammatory factors were also dose-dependently down-regulated (*p* < 0.05, Figures 2D–G). Furthermore, the Western blot experiment determined the protein expression of the AMPK/SIRT1 pathway. Compared with control group, the AMPK/SIRT1 protein expression was considerably down-regulated in H9C2 cells and Primary cardiomyocytes when subjected to OGD, while the protein expression of AMPK/SIRT1 pathway was upregulated in a dose-dependent manner after the intervention of Arctigenin at different concentration (compared with OGD group) (*p* < 0.05, Figure 2H). The above findings indicated that Arctigenin dose-dependently inhibited the OGD-mediated oxidative stress level and inflammatory response in cardiomyocytes *in vitro*.

### Repressing AMKP Phosphorylation Reversed Arctigenin-Mediated Protective Effects in Cardiomyocytes

**FIGURE 3:**
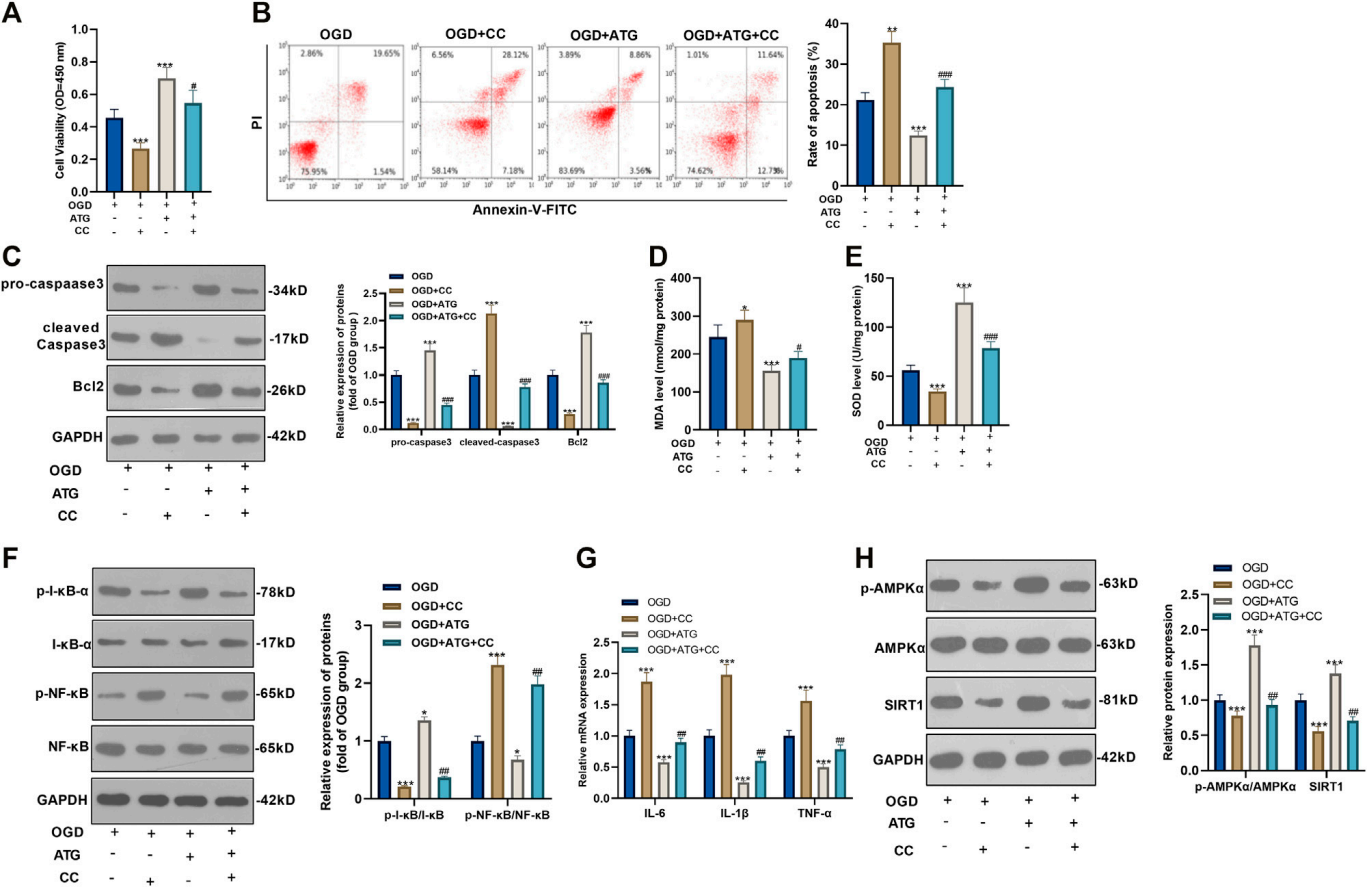
Repressing AMKP phosphorylation reversed Arctigenin-mediated protective effects in cardiomyocytes. 100 μM Arctigenin was given to treat OGD-mediated H9C2 cells, and on the basis of this, 1 nM AMPK inhibitor Compound C was given for intervention. **(A, B)** MTT method and flow cytometry were used to detect cell viability and apoptosis. **(C)** Western blot detection of Caspase-3 and Bcl2 expression. **(D, E)** RT-PCR was used to evaluate the content of oxidative stress markers MDA and SOD. **(F, G)** Western blot and RT-PCR experiments were performed for the protein expression of NF-κB/I-κB and the levels of inflammatory factors IL-6, IL-1β and TNF-α. **(H)** Western blot assay to detect the protein expression of the AMPK/SIRT1 pathway. ***p < 0.001 (vs. OGD group), #*p* < 0.05, ##*p* < 0.01, ###*p* < 0.001 (vs. OGD+ Arctigenin group), *N* = 5.

To ensure the role of AMPK/SIRT1 in Arctigenin induced protective effects in cardiomyocytes, we treated OGD-mediated H9C2 cells with 200 µM Arctigenin and 1 nM AMPK inhibitor Compound C. Then, the MTT method and flow cytometry were taken respectively to detect the cell vitality and apoptosis. It turned out that after intervention with Compound C, the cell vitality decreased notably, and cell apoptosis was increased (*p* < 0.05, Figures 3A,B). The expression of the apoptotic protein was further verified via Western blot. The outcomes represented that the activity of pro-apoptotic protein Caspase-3 was increased after the addition of Compound C, while the expression of anti-apoptotic protein Bcl2 was decreased (*p* < 0.05, Figure 3C). Next, we examined the content of oxidative stress markers (MDA and SOD) and found that compared with the OGD+ Arctigenin group, MDA content in myocardial cells was upregulated after Compound C intervention, while SOD activity was decreased (*p* < 0.05, Figures 3D,E). In the meantime, Western blot and RT-PCR were conducted to assess the protein expression of I-κB/NF-κB and the levels of inflammatory factors. It was demonstrated that compared with the OGD+ Arctigenin group, the intervention of Compound C upregulated p-NF-κB expression but the down-regulated p-I-κB expression, and the expression of inflammatory factors were also significantly increased (*p* < 0.05, Figures 3F,G). Additionally, Western blot results illustrated that the AMPK/SIRT1 pathway’s protein expression was notably reduced after the addition of Compound C (*p* < 0.05, Figure 3H). The above experimental results revealed that Arctigenin alleviated OGD-mediated myocardial cell injury by activating the AMPK/SIRT1 pathway.

### Repressing SIRT1 Reversed Arctigenin-Mediated Protective Effects in Cardiomyocytes

**FIGURE 4:**
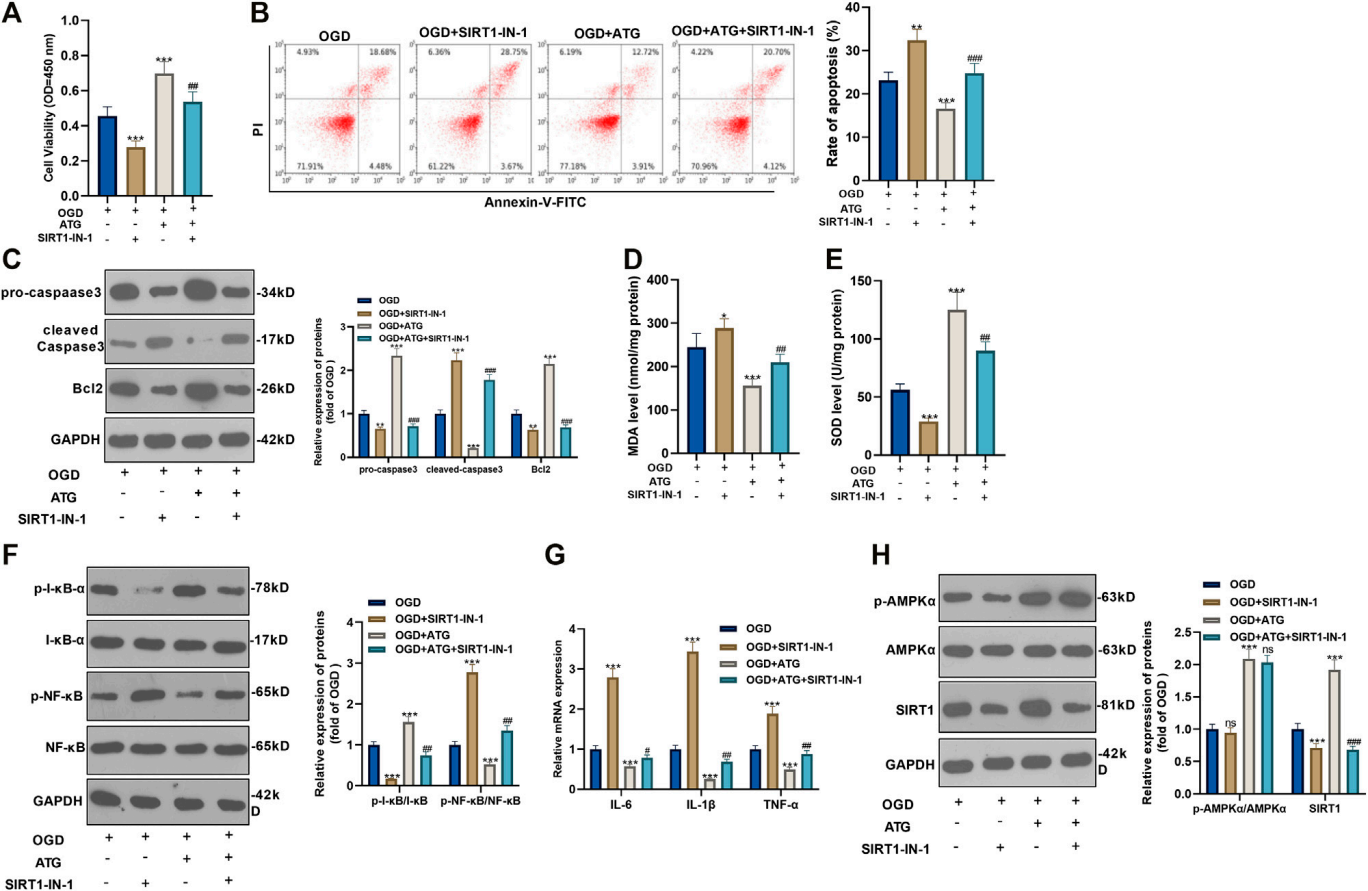
Repressing SIRT1 reversed Arctigenin-mediated protective effects in cardiomyocytes. 100 μM Arctigenin was given to treat OGD-mediated H9C2 cells, and on the basis of this, 0.2 μM SIRT1 inhibitor SIRT1-IN-1 was given for intervention. **(A, B)** MTT method and flow cytometry were used to detect cell viability and apoptosis. **(C)** Western blot detection of Caspase-3 and Bcl2 expression. **(D, E)** RT-PCR was used to evaluate the content of oxidative stress markers MDA and SOD. **(F, G)** Western blot and RT-PCR experiments were performed for the protein expression of NF-κB/I-κB and the levels of inflammatory factors IL-6, IL-1β and TNF-α. **(H)** Western blot assay to detect the protein expression of the AMPK/SIRT1 pathway. **p < 0.01, ***p < 0.001 (vs. OGD group), #*p* < 0.05, ##*p* < 0.01, ###*p* < 0.001 (vs. OGD+ Arctigenin group), *N* = 5.

To ensure the role of AMPK/SIRT1 in Arctigenin induced protective effects in cardiomyocytes, we treated OGD-mediated H9C2 cells with 200 µM Arctigenin and 1 nM SIRT1 inhibitor SIRT1-IN-1. Then, the MTT method and flow cytometry were taken respectively to detect the cell vitality and apoptosis. It turned out that after intervention with SIRT1-IN-1, the cell vitality decreased notably, and cell apoptosis was increased (*p* < 0.05, Figures 4A,B). The expression of the apoptotic protein was further verified via Western blot. The outcomes represented that the activity of pro-apoptotic protein Caspase-3 was increased after the addition of SIRT1-IN-1, while the expression of anti-apoptotic protein Bcl2 was decreased (*p* < 0.05, Figure 4C). Next, we examined the content of oxidative stress markers (MDA and SOD) and found that compared with the OGD+ Arctigenin group, MDA content in myocardial cells was upregulated after SIRT1-IN-1 intervention, while SOD activity was decreased (*p* < 0.05, Figures 4D,E). In the meantime, Western blot and RT-PCR were conducted to assess the protein expression of I-κB/NF-κB and the levels of inflammatory factors. It was demonstrated that compared with the OGD+ Arctigenin group, the intervention of SIRT1-IN-1 upregulated p-NF-κB expression but the down-regulated p-I-κB expression, and the expression of inflammatory factors were also significantly increased (*p* < 0.05, Figures 4F,G). Additionally, Western blot results illustrated that the SIRT1 pathway’s protein expression was notably reduced after the addition of SIRT1-IN-1, but nor for p-AMPKα (*p* < 0.05, Figure 4H). The above experimental results revealed that Arctigenin alleviated OGD-mediated myocardial cell injury by activating the AMPK/SIRT1 pathway.

### Arctigenin Improved Heart Functions in AMI/R-Rats *in vivo*

**FIGURE 5:**
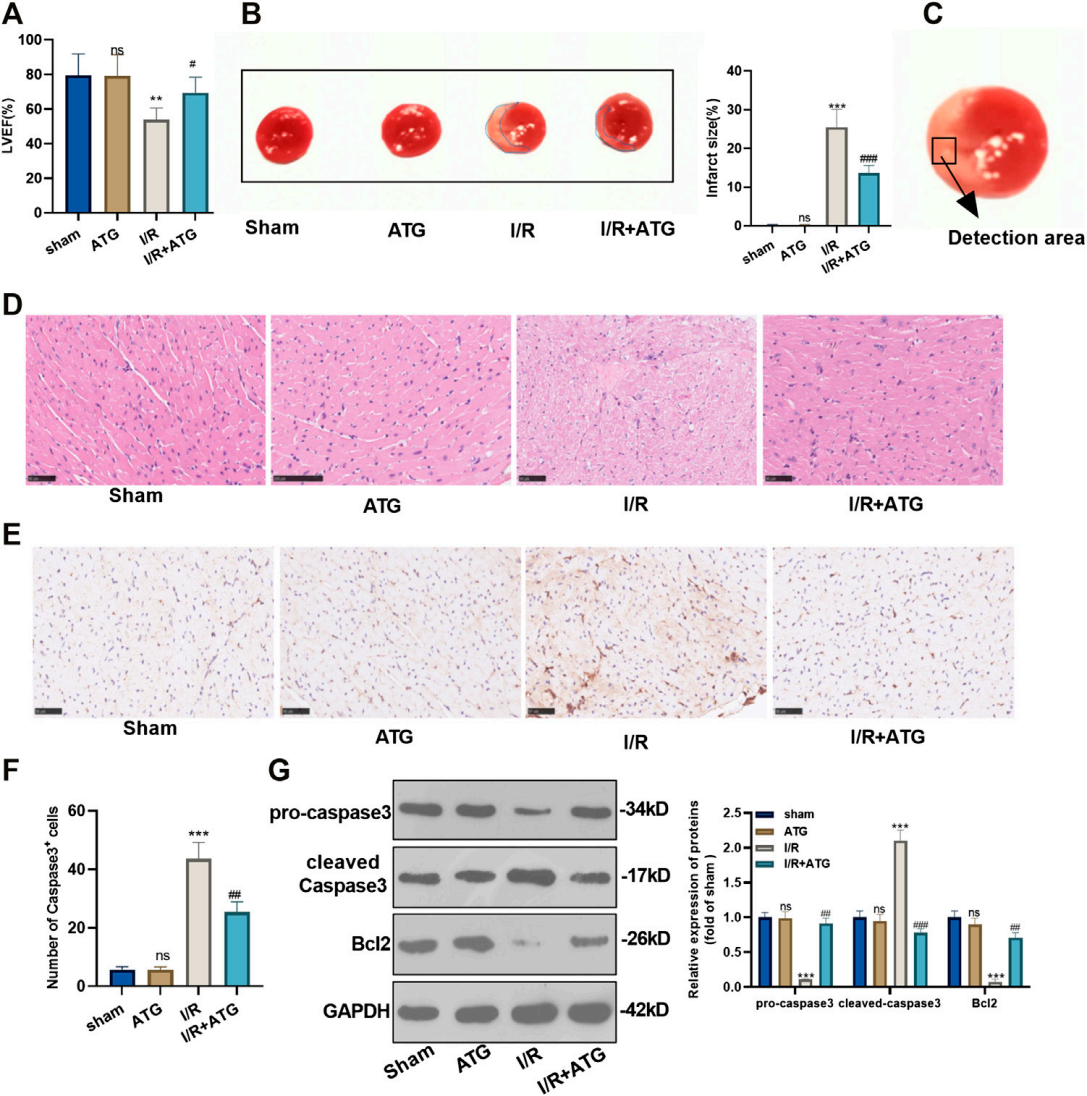
Arctigenin remarkably mitigated the AMI/R-induced myocardial damage *in vivo*. The LAD permanent ligation method was performed to establish a rat MI model *in vivo*, and Arctigenin was given (100 µmol/kg, intraperitoneal injection), and the heart tissue was taken on the 21st day. **(A)** Change of LVEF of rats. **(B)** TTC staining was used to detect the AMI/R area in each group. **(C)** The detected area was shown. **(D)** HE staining explored the arrangement of myocardial fibrous tissue and inflammatory cell infiltration. **(E)** and **(F)** The number of Caspase3 positive cells was examined via immunohistochemistry. **(G)** Western blot test detection for the expression of apoptosis-related proteins caspase3 and Bcl2. nsP >0.05, ***p < 0.001 (vs.sham group), #*p* < 0.05, ##*p* < 0.01, ###*p* < 0.001 (vs.IR group), *N* = 5.

To explore how Arctigenin affects the myocardial tissue of MI rat *in vivo*, we took the rats’ heart tissues after the 21 days of their LAD ligation and performed echocardiography. It was found that the addition of 100 µmmol/kg Arctigenin alone showed no apparent changes of heart functions, which proved that Arctigenin was non-toxic *in vivo*. Then, the rat AMI/R model was built and treated with Arctigenin (100 µmmol/kg). It turned out that the LVEF of AMI/R rat was reduced compared with that of sham group rat. After the therapy of Arctigenin, the LVEF of AMI/R rat significantly increased (*p* < 0.05, Figure 5A). Moreover, TTC staining was applied to measure the AMI/R area in the rats, and it was found that after treatment with 100 µmmol/kg of Arctigenin, there was no significant difference in infarct size compared with healthy myocardial tissue, but the infarct area of the myocardium in the AMI/R group was considerably increased. Next, Arctigenin was given for intervention. The infarct size decreased markedly (*p* < 0.05, Figure 5B). Further, histopathological examination was used to evaluate the pathological changes of ischemic lesions (shown in Figure 5C). HE staining results illustrated that the myocardial fibers in the sham operation group and the Arctigenin group were neatly arranged without inflammatory cell infiltration. In the AMI/R group, myocardial fibers were disordered with obvious inflammatory cell infiltration, but compared with the AMI/R group, the arrangement of myocardial fiber tissue and inflammatory cell infiltration were improved with the Arctigenin treatment obviously relieved myocardial damage and inflammatory cell infiltration (*p* < 0.05, Figure 5D). Immunohistochemical results also showed no significant difference in the number of positive Caspase3 cells in the Arctigenin group, while the number of positive Caspase3 cells in the AMI/R group was raised compared with the sham group. On this basis, Arctigenin’s intervention a reversed the effects induced by AMI/R (*p* < 0.05, Figures 5E,F). Finally, Western blot assay was carried out to detect Caspase3 and Bcl2 expression. The results demonstrated no significant difference in the expression of apoptosis-related proteins in ATG group, while in the AMI/R group, the expression of pro-apoptotic protein Caspase3 was increased and the expression of anti-apoptotic protein Bcl2 was down-regulated (compared with the sham group). However, after the addition of Arctigenin, Caspase3 expression was down-regulated at a concentration gradient, and Bcl2 expression was upregulated (*p* < 0.05, Figure 5G). These findings manifested that Arctigenin improved the injury of rats’ heart function *in vivo*.

### Arctigenin Attenuated Oxidative Stress and Inflammation After AMI/R

**FIGURE 6:**
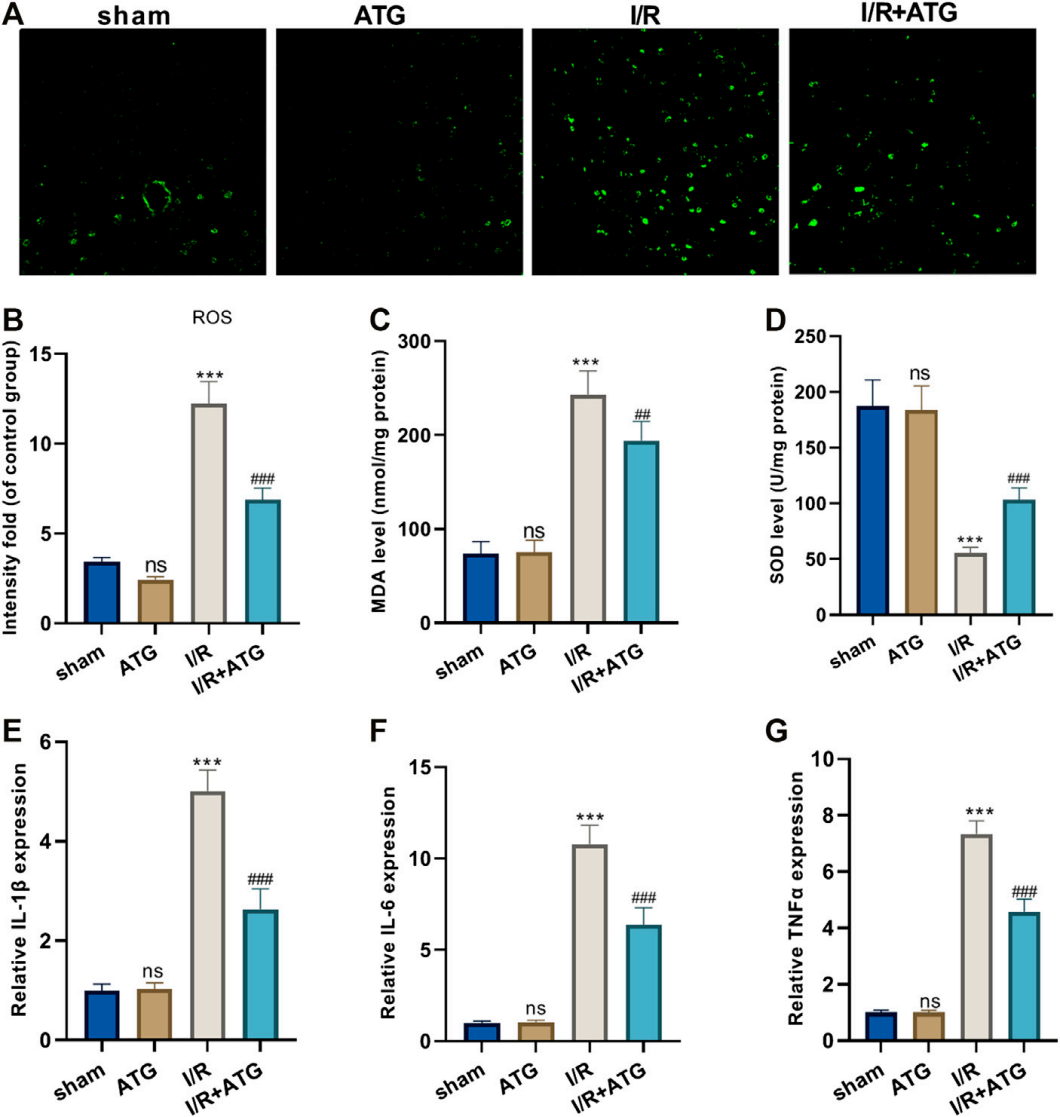
Arctigenin reduced oxidative stress and inflammation in AMI/R rats *in vivo*. The MI model was constructed, and treated with 100 µmol/kg of Arctigenin. The cardiac tissue was taken on the 21st day after surgery. **(A–D)** ROS level and the content of oxidative stress markers MDA and SOD in rat myocardium. **(E–G)** RT-PCR experiment to detect the levels of inflammatory factors IL-6, IL-1β, TNF-α. nsP >0.05, ***p < 0.001 (vs.sham group), ##*p* < 0.01, ###*p* < 0.001 (vs.IR group), *N* = 5.

For the more profound exploration of how Arctigenin affects cardiac oxidative stress levels of MI rats, we utilized the DCFH-DA fluorescent dyes to measure the ROS levels in the myocardial tissue and tested the content of oxidative stress markers (MDA and SOD). It was found that compared with the sham group, there was no significant difference in the level of ROS and the content of MDA and SOD after the treatment of 100 μmol/kg Arctigenin. Under AMI/R, the ROS level and MDA content in the AMI/R group was increased, and the SOD activity was reduced. However, Arctigenin restrained the ROS level and MDA content, while enhanced SOD activity (*p* < 0.05, Figures 6A–D). Besides, RT-PCR experiments were performed to measure the levels of inflammatory factors, the results showed that Arctigenin intervention didn’t alter their expression in the normal rats, but repressed them in the AMI/R rats (*p* < 0.05, Figures 6E–G). Those results indicated that Arctigenin attenuates the oxidative stress level and inflammatory response of the AMI/R rat.

### Arctigenin Activated the AMPK/SIRT1 Pathway

**FIGURE 7:**
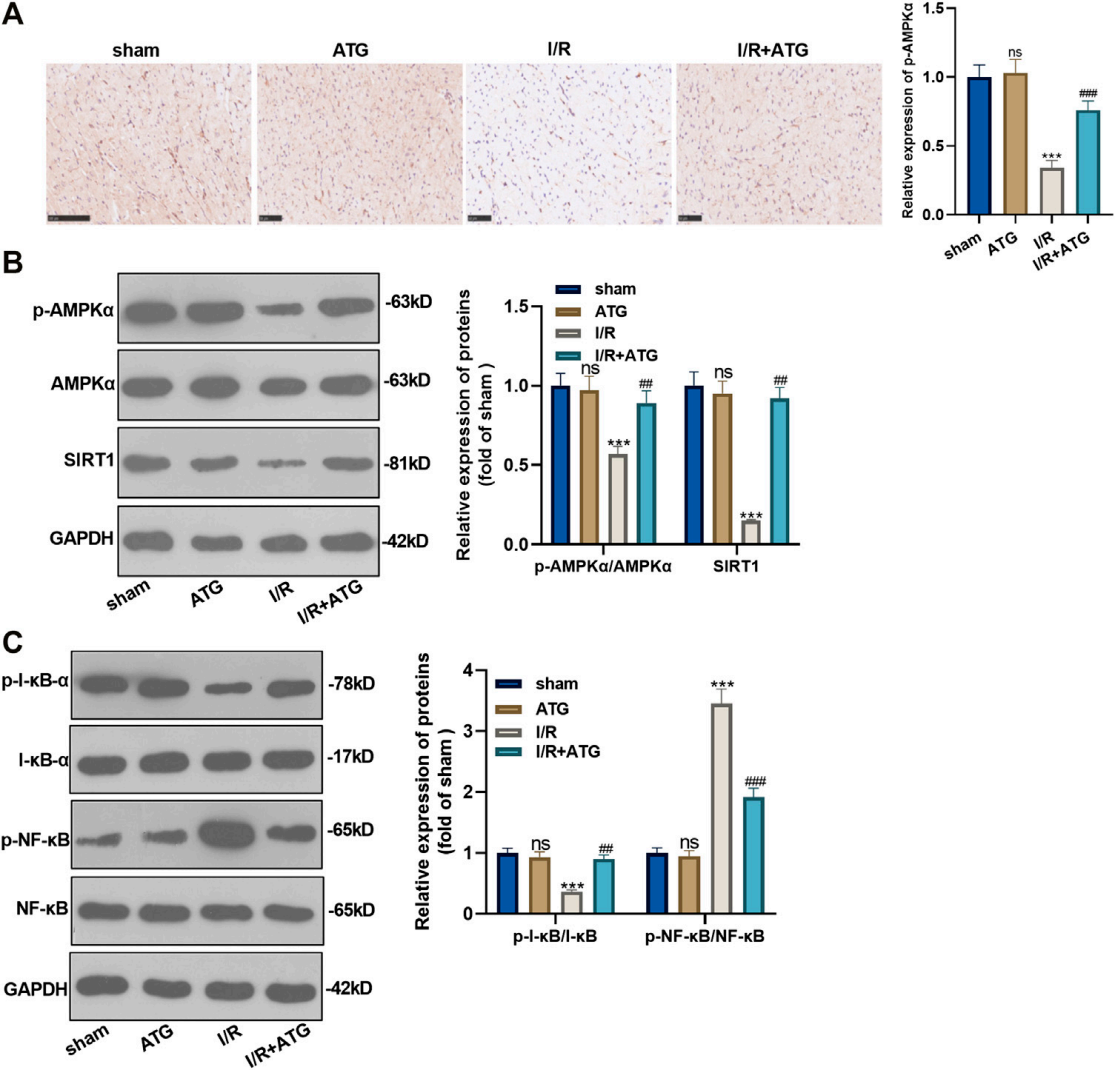
Arctigenin activated the AMPK/SIRT1 signaling pathway. The MI model was constructed, and treated with 100 µmol/kg of Arctigenin. The cardiac tissue was taken on the 21st day after surgery. **(A)** Immunohistochemistry detection of AMPK expression in the heart tissues. **(B, C)** Western blot experiments were used to assess the protein expression of AMPK/SIRT1 pathway and downstream pathway NF-κB/I-κB. nsP >0.05, ***p < 0.001 (vs.sham group), ###*p* < 0.001 (vs.IR group), *N* = 5.

**FIGURE 8:**
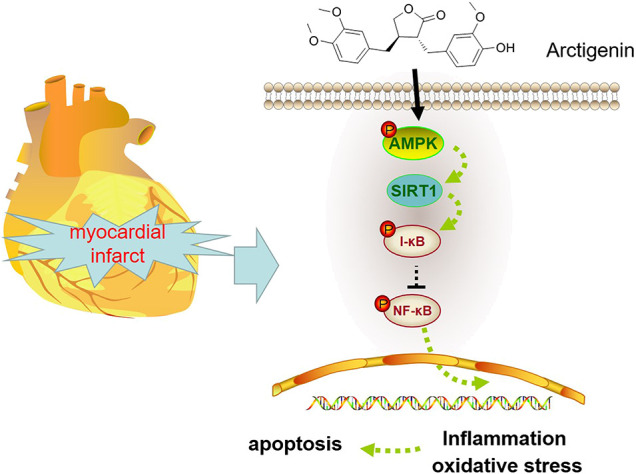
Mechanism diagram Arctigenin activates the AMPK/SIRT1 signaling pathway to upregulate the expression of I-κB and inhibit the expression of NF-κB, thereby reducing oxidative stress, inflammation and apoptosis in MI rats.

We applied the immunohistochemical test to detect the expression of AMPK, and it was found that the expression of AMPK in the Arctigenin group had no significant difference compared with the sham group. However, AMI/R induced inhibited expression of AMPK. Treatment with Arctigenin notably promoted AMPK in the myocardial tissues (*p* < 0.05, Figure 7A). Then, Western blot was taken to examine the protein expression of the AMPK/SIRT1 and NF-κB/I-κB. The results revealed that AMI/R led to lower level of phosphorylated AMPK, SIRT1, and phosphorylated I-κB, but promoted NF-κB phosphorylation (Figures 7B,C). After the Arctigenin intervention, AMPK/SIRT1 pathway became significantly, while NF-κB phosphorylation level was down-regulated and I-κB phosphorylation level was upregulated (*p* < 0.05, Figures 7B,C). Collectively, the above data revealed that Arctigenin relieved AMI/R via promoting the activation of the AMPK/SIRT1 pathway (Figure 8).

## Discussion

More and more evidence show that inflammatory response and oxidative stress make a great contribution to the progression of atherosclerosis and secondary injury after myocardial injury, and it is a crucial mediator of adverse cardiac remodeling after MI (Marchant et al., 2012; Zhu et al., 2018; Ziegler et al., 2018; Yan et al., 2019). Apoptosis is a kind of continuous programmed cell death that maintains the tissue homogenization state. A large number of studies have found that prolonged myocardial ischemia can lead to increased necrosis, which accelerates the development of myocardial apoptosis and determines the degree of myocardial damage (Wu et al., 2018). More importantly, OGD induced oxidative stress is closely related to cell apoptosis. Studies have shown that excessive ROS can damage the myocardial mitochondrial structure, thus triggering mitochondria-mediated apoptosis (Zorov et al., 2014). This paper focused on the role of AMPK-SIRT1 pathway mediated by Arctigenin on the oxidative stress and pro-inflammatory cytokine response after AMI/R. It was found that Arctigenin alleviated oxidative stress and inflammatory response by activating the AMPK/SIRT1 pathway, thus playing a myocardial protective role.

Arctigenin has attracted much attention due to its important biological and physiological roles. For instance, Arctigenin relieved ultraviolet A (UVA) irradiation induced damage to the stemness of human mesenchymal stem cells (hMSCs) derived from adipose tissue. The underlying mechanism involves reversing hypoxia-inducible factor (HIF)-1α expression, reducing PGE2 production through the inhibition of MAPKs (p42/44 MAPK, p38 MAPK, and JNK) and NF-κB (Park et al., 2018). In the In a mouse model of depression, repeated Arctigenin treatment shows antidepressant- and anxiolytic-like effects via promoting the expressions of angiogenin (ANG), thrombopoietin (TPO), and vascular endothelial growth factor (VEGF) (Du et al., 2019). Interestingly, increasing studies have proved that Arctigenin exerts a potential therapeutic drug in cardiovascular diseases. For example, in the aconitine-induced rat model, Arctigenin significantly delayed the arrhythmia onset of the rat model by regulating multi-ion channels (Zhao et al., 2013). In the myocardial ischemia/reperfusion (MI/R) model, the arrhythmia of rats could be reversed by Arctigenin by activating Nrf2 signaling pathway (Yang et al., 2018). In another study, Arctigenin exhibits antioxidative and anti-inflammatory effects in a rat model of AMI through regulation of the iNOS, COX-2, ERK1/2, and HO-1 pathways (Zhang and Yang, 2018). Similarly, we established a rat model of MI *in vivo* and *in vitro* in this study using OGD and LAD permanent ligation method. Arctigenin was found to ameliorate myocardial cell apoptosis through repressing I-κB-NF-κB pathway, which further confirmed the anti-inflammation and antioxidant effect of Arctigenin.

Previous studies have suggested that Arctigenin could activate AMPK pathway, thereby improving multiple diseases (Han et al., 2016; Meng et al., 2017; Alves-Fernandes and Jasiulionis, 2019). AMPK, a member of the Serine/threonine (Ser/Thr) kinase group, is widely distributed in various cells and organs as one of the central regulators of metabolism in eukaryotic cells and organisms (Jiang et al., 2018). Adiponectin (APN) was found to reduce oxidative stress and NLRP3 inflammasome activation after cerebral ischemia-reperfusion injury by regulating AMPK/GSK-3 (Liu et al., 2020). Additionally, Yu J et al. also found that Ezetimibe (Eze) reduces oxidative stress and subsequent neuroinflammatory response by activating AMPK/Nrf2/TXNIP pathways in MCAO rats (Yu et al., 2020). These findings manifest that AMPK activation plays a protective role in ischemic brain injury. What’s more, AMPK activation is also important in myocardial ischemic disease. Studies have shown that ginsenoside Rg3 protects the heart from isoproterenol (ISO)-induced MI by activating AMPK-mediated autophagy (Sun et al., 2020). At the same time, melatonin improves mitochondrial biogenesis by activating the AMPK/PGC1 pathway, thereby reducing myocardial injury caused by ischemia/reperfusion (Qi and Wang, 2020). This study found that the expression of AMPK in the MI group decreased comparing the sham group. Under Arctigenin’s intervention, phosphorylated AMPK level was notably increased. Meanwhile, Compound C, an AMPK inhibitor, was given to intervene AMPK in cardiomyocytes. It was found that cell viability was reduced, and apoptosis increased after adding Compound C in the Arctigenin treated group. Moreover, the SOD activity was significantly reduced, MDA content and the expression of inflammatory factors were considerably increased. This is consistent with the above conclusion that Arctigenin exerted its effects via inducing AMPK activation.

SIRT1 is a deacetylase that plays an important role in the control of many cell processes, and its down-regulation is associated with various metabolic abnormalities (Shokri Afra et al., 2019). Several studies have shown that SIRT1 is also of outstanding value in myocardial ischemic disease. For example, Zhang X et al. reported that Dex inhibits myocardial ischemia/reperfusion injury by activating the SIRT1/mTOR axis (Zhang et al., 2020). Meanwhile, Shu L et al. illustrated that lncRNA ANRIL promoted the up-regulation of SIRT1 expression by down-regulating miR-7-5p, thus playing a protective role in H9C2 myocardial cell injury induced by hypoxia (Shu et al., 2020). In recent decades, many studies have shown that AMPK/STRT1 plays a significant role in myocardial injury or apoptosis and inflammatory response. Previous studies have also reported that catechins attenuate TNFα-induced inflammation in 3T3-L1 adipocytes through the AMPK-SIRT1 pathway (Cheng et al., 2019). Moreover, the natural compound pineoleic acid (PLA) isolated from pine seed kernel oil improved oleacid-induced fat generation and oxidative stress through the AMPK/SIRT1 pathway in HepG2 cells (Zhang et al., 2019). Meanwhile, Gu X et al. also found that resveratrol inhibited alcohol-induced inflammatory response, oxidative stress and caspase-3 activity in rats by inhibiting AMPK/SIRT1/P38 pathway (Gu et al., 2018). Those evidence suggest that AMPK/SIRT1 pathway has important value in myocardial injury. On the other hand, the fact that SIRT1 functions in ischemic heart disease by regulating its downstream NF-κB/I-κB pathway has been reported. Li D and others discovered that caffeic acid phenylethyl ester (CAPE) inhibit the NF-κB signaling by promoting SIRT1/eNOS expression, thus inhibiting oxidative stress, inflammation, fibrosis, and necrosis and improve the rat myocardial ischemia damage (Li et al., 2018). Another study reported that punicalagin inhibits NF-κB nuclear translocation by activating the SIRT1-mediated NRF-2-HO-1 signaling pathway, thereby relieving myocardial ischemia/reperfusion injury-induced cardiac oxidative stress and inflammation (Yu et al., 2019). Encouragingly, this study also found that Arctigenin plays a significant role in cardiac protection from oxidative stress and inflammatory injury in rats by activating AMPK/SIRT1 signaling pathways. After inhibiting the AMPK phosphorylation, SIRT1 level was significantly decreased, accompanied with re-activated NF-κB signaling pathway. Hence, those data further indicate that Arctigenin myocardial protection in MI rats via AMPK/SIRT1. This fact is consistent with the reports of Huang J (Huang et al., 2019) and Tian L (Tian et al., 2019) et al.

To sum up, through functional experiments, it was found that the intervention of Arctigenin inhibited the apoptosis of cardiomyocytes induced by MI with anti-oxidative stress and anti-inflammatory effects by activating the AMPK/SIRT1 pathway. This finding provides a new reference direction for the treatment and prognosis improvement of MI. However, further experiments are needed to confirm our conclusion: 1) genetic interventions on AMPK and SIRT1 to verify whether Arctigenin exerts its effect dependently through AMPK/SIRT1 pathway; 2) *in vivo* experiment to demonstrate that Arctigenin plays a role in reducing cardiomyocyte damage induced by MI.

## Data Availability Statement

The datasets presented in this study can be found in online repositories. The names of the repository/repositories and accession number(s) can be found in the article/Supplementary Material.

## Ethics Statement

The animal study was reviewed and approved by Ethics Review Board of Renmin Hospital of Wuhan University.

## Author Contributions

Conceived and designed the experiments: X-JJ, C-YL, and YZ; Performed the experiments: C-YL, YZ; Statistical analysis: C-YL, YZ, TC, and J-YL; Wrote the paper: C-YL. All authors read and approved the final manuscript.

## Funding

This research did not receive any specific grant from funding agencies in the public, commercial, or not- for- profit sectors.

## Conflict of Interest

The authors declare that the research was conducted in the absence of any commercial or financial relationships that could be construed as a potential conflict of interest.
